# The NAC transcription factor MdNAC29 negatively regulates drought tolerance in apple

**DOI:** 10.3389/fpls.2023.1173107

**Published:** 2023-07-06

**Authors:** Sen Li, Xiuli Jing, Qiuping Tan, Binbin Wen, Xiling Fu, Dongmei Li, Xiude Chen, Wei Xiao, Ling Li

**Affiliations:** ^1^ College of Horticulture Science and Engineering, Shandong Agricultural University, Tai’an, China; ^2^ State Key Laboratory of Crop Biology, Shandong Agricultural University, Tai’an, China; ^3^ Shandong Collaborative Innovation Center for Fruit and Vegetable Production with High Quality and Efficiency, Shandong Agricultural University, Tai’an, China

**Keywords:** apple, MdNAC29, MdDREB2A, drought tolerance, F-box protein, RNA-seq

## Abstract

Drought stress is an adverse stimulus that affects agricultural production worldwide. NAC transcription factors are involved in plant development and growth but also play different roles in the abiotic stress response. Here, we isolated the apple *MdNAC29* gene and investigated its role in regulating drought tolerance. Subcellular localization experiments showed that *MdNAC29* was localized to the nucleus and transcription was induced by the PEG treatment. Over-expression of *MdNAC29* reduced drought tolerance in apple plants, calli, and tobacco, and exhibited higher relative conductivity, malondialdehyde (MDA) content, and lower chlorophyll content under drought stress. The transcriptomic analyses revealed that *MdNAC29* reduced drought resistance by modulating the expression of photosynthesis and leaf senescence-related genes. The qRT-PCR results showed that overexpression of *MdNAC29* repressed the expression of drought-resistance genes. Yeast one-hybrid and dual-luciferase assays demonstrated that MdNAC29 directly repressed *MdDREB2A* expression. Moreover, the yeast two-hybrid and bimolecular fluorescence complementation assays demonstrated that MdNAC29 interacted with the MdPP2-B10 (F-box protein), which responded to drought stress, and MdPP2-B10 enhanced the repressive effect of MdNAC29 on the transcriptional activity of the *MdDREB2A*. Taken together, our results indicate that *MdNAC29* is a negative regulator of drought resistance, and provide a theoretical basis for further molecular mechanism research.

## 1 Introduction

Plants are exposed to a variety of adverse environmental stressors in their natural habitats, including biotic stressors (fungal, bacterial, viral, and insect pest) and abiotic stressors (high salinity, drought, flooding, heat, cold, and injury) (Zhang et al., 2012). Abiotic stressors are significant during plant growth and development, particularly in the face of global warming, limited water supplies, and soil desertification. Among the adverse external stimuli, drought stress has become a major environmental factor limiting crop productivity and management (Zhu and Ni, 2016; Basia and Arie, 2005). When plants are subjected to drought stress, they regulate gene expression and produce new proteins through the perception and transmission of drought signals, resulting in several morphological, physiological, and biochemical changes to ensure normal development and growth (Kavi et al., 2005).

NAC (NAM-ATAF1/2-CUC2) is one of the largest transcription factor (TF) families in plants and is widely distributed in terrestrial plants (Puranik et al., 2012; An et al., 2018). The first NAC TF was cloned from Petunia (Souer et al., 1996) and subsequently found in *Arabidopsis* and rice. NAC TFs share a highly conserved NAC domain of about 150 amino acids (aa) at the N-terminal end and a highly variable transcriptional regulatory region at the C-terminal end (Olsen et al., 2005). Studies have shown that NAC TFs are involved in the regulation of plant growth and development (Sun et al., 2012; Wen et al., 2022). In apples, MdNAC52 regulates the biosynthesis of anthocyanin and proanthocyanidin through *MdMYB9* and *MdMYB11* (Sun et al., 2019). In *Arabidopsis*, *AtNAC029* is involved in leaf senescence, and overexpression of *AtNAC029* causes premature senescence (Ma et al., 2020). NAC TFs play an important regulatory role in the abiotic stress response (Shao et al., 2015). Drought, salt stress, or abscisic acid (ABA) induce the expression of *OsNAC5* and *OsNAC10* in rice and *AtAF1*, *ANAC19*, *ANAC55*, and *ANAC72* in *Arabidopsis*, demonstrating the role of these NAC TFs in drought and salt resistance (Tran et al., 2004; Lu et al., 2007; Jeong et al., 2010; Takasaki et al., 2010). The expression level of wheat *TaNAC2* increases significantly under drought stress and enhances drought tolerance by activating drought stress response genes (Mao et al., 2014). Overexpression of *OsNAC106* reduces salt stress tolerance in rice and the deletion mutation results in increased salt tolerance (Sakuraba et al., 2015). In summary, NAC TFs have different functions in response to abiotic stressors.

Other TF families, including AP2/ERF, MYB, TCP, AREB, and WRKY play important roles in abiotic stress (Li et al., 2020; Li et al., 2021b). DREBs are a subfamily of AP2/EREBP TFs that have a conserved AP2 domain structure. The DREB gene has been extensively studied during abiotic stress in plants (Agarwal et al., 2006; Khan, 2014; Eckardt, 2019). DREB2A members are mainly involved in drought and salt stress. Over-expressing the wheat *TaDREB2* gene increases the survival of wheat under severe drought conditions (Morran et al., 2011). Over-expressing *PgDREB2A* results in better tolerance to high salt and drought stress in tobacco (Agarwal et al., 2010). Overexpressing *MdDREB2A* in apple calli enhances drought tolerance (Lian et al., 2021). In addition, *MdAREB1A*, *MdRD22*, *MdRD29A*, and *MdRD29B* are responsive to drought or osmotic stress in apples (Ma et al., 2017; An et al., 2018; Shao et al., 2019).

F-box proteins contain an F-box motif and have substrate recognition properties during ubiquitin-mediated protein hydrolysis. These proteins are called F-box because the motif was first identified in *CyclinF* (Risseeuw et al., 2003). Several studies have shown that F-box proteins play important roles in various signaling pathways, such as stress resistance, photomorphogenesis, development of flower organs, regulation of circadian rhythms, and growth hormone signaling (Sonneveld et al., 2005; Zhang et al., 2008; Sheard et al., 2010; Koops et al., 2011). Apple F-box protein MdAMR1L1 regulates the degradation of the ascorbic acid (Asc) synthesis catalase MdGMP1 protein through the ubiquitination pathway, affecting Asc synthesis and thus fruit quality and stress tolerance (Ma et al., 2022). KUF1 (KARRIKIN-upregulated F-box protein) negatively regulates drought tolerance in *Arabidopsis* by modulating various physiological traits, morphological adjustments, and ABA responses (Dubois, 2022; Tian et al., 2022). Overexpression of the F-box protein AtPP2-B11 reduces drought tolerance in *Arabidopsis* (Li et al., 2014). The soybean F-box-Like protein GmFBL144 interacts with a small heat shock protein to negatively regulate plant drought stress tolerance (Xu et al., 2022). Loss-of-function of ARABIDOPSIS F-BOX PROTEIN HYPERSENSITIVE TO ABA 1 enhances drought tolerance and delays germination (Kim et al., 2021).

Apples are important cultivated fruit trees in China. Apples often suffer from drought stress during growth, which seriously limits fruit quality and yield. NAC TFs play an important role in the apple response to abiotic stress. *MdNAC29* plays a role in cold tolerance (An et al., 2018); however, its role in drought tolerance in apples has not been investigated. In this study, we cloned the NAC TF *MdNAC29* (Phytozome Gene accession number: MDP0000481448) from apples. Over-expressing *MdNAC29* reduced drought tolerance. The quantitative real-time polymerase chain reaction (qRT-PCR) results showed that overexpressing *MdNAC29* reduced drought resistance by repressing the expression of drought tolerance genes. The yeast-one-hybrid (Y1H) and luciferase (Luc) assays demonstrated that *MdNAC29* negatively regulates drought tolerance by directly binding to the *MdDREB2A* promoter and repressing its expression. Furthermore, MdNAC29 interacted with the F-box protein MdPP2-B10. Further studies showed that MdPP2-B10 responded to drought stress and enhanced the repressive effect of MdNAC29 on the transcriptional activity of the *MdDREB2A*. Our study provides new insight into the role of *MdNAC29* in regulating drought tolerance in apples.

## 2 Materials and methods

### 2.1 Plant materials and growth conditions

The plant materials used in this study included ‘GL-3’ (*Malus domestica*) apple tissue culture seedlings (Wen et al., 2022), ‘Orin’ apple calli, and tobacco (*Nicotiana benthamiana*) seeds. The ‘GL-3’ apple seedlings were cultured in an incubator (16 h-light/8 h-dark) at 24°C. The apple calli were cultured on MS medium supplemented with 0.5 mg/L 6-BA and 1.5 mg/L 2,4-D at 24°C in the dark and subcultured at 12–16 day intervals. The sterilized tobacco seeds were planted on MS medium and incubated in the dark at 4°C for 72–96 h, then transferred to an incubator (incubation conditions: 14 h-light/10 h-dark; temperature: 24/22°C). The apple calli and tissue-cultured tobacco were used for genetic transformation. The substrate-cultivated transgenic tobacco and apple seedlings were used for functional identification.

### 2.2 RNA extraction and real-time quantitative PCR analysis

Total RNA was extracted using the FastPure^®^ Plant Total RNA Isolation Kit-RC401 (Vazyme, Nanijing, China). RNA integrity was examined by agarose gel electrophoresis. Using RNase-Free ddH_2_O as the control, 1 µL of RNA solution was pipetted onto a NanoPhotometer P-360 ultra-microspectrophotometer (Munich, Germany) for concentration and purity, A260/A280 was in the range of 1.8-2.0, indicating that a high-quality RNA solution was obtained. Reverse transcription was performed using the HiScript III RT SuperMix kit R323-01 (Vazyme) to obtain cDNA. qRT-PCR was performed according to the fluorescent dye provided in the Ultra SYBR Mixture RT-qPCR CW0957 Kit (CW Biotech, Taizhou, China). The comparative Ct (2^–ΔΔCt^) method was used for analysis. *MdACTIN* was the internal apple reference gene (An et al., 2018), and qRT-PCR primers are listed in **Supplementary Table 1**.

### 2.3 Subcellular localization analysis

*MdNAC29* was constructed on the *PRI101-GFP* vector and the recombinant plasmid was transferred into GV3101 *Agrobacterium*. Onion *(Allium cepa)* bulbs were selected and the inner epidermis was cut into multiple rectangular square pieces with a sterile scalpel blade. The pre-cultured onion inner epidermis was added to the prepared infection solution and infiltrated at 150 rpm for 20 min at 28°C. The excess bacterial solution was blotted off the inner epidermis with sterile blotting paper and re-laid on new MS solid medium on an ultra-clean bench. The inner onion epidermis was placed on a slide, and fluorescence of the target fusion protein was observed under a scanning confocal microscope LSM-880 (Zeiss AG, Zena, Germany) and photographed.

### 2.4 Vector construction and plant transformation

The *MdNAC29* coding sequence (CDS) was ligated into the *PRI101* vector (35S promoter, GFP) to obtain the *MdNAC29* overexpressing vector and then transformed into *Agrobacterium* LBA-4404. Good-growing ‘GL-3’ apple tissue cultures seedlings were selected, the leaf tips and petioles were removed, the leaves were scratched vertically on the veins, laid flat on pre-culture medium, and incubated for 2-3 d protected from light. The leaves were incubated overnight at 220 rpm and 28°C, then 1 mL of activated bacteria was added to 30 mL of YEP+Rif+Kan liquid medium and shaken until the OD_600_ nm = 0.6-0.8. The mixture was centrifuged and the supernatant was discarded. A 30 mL portion of the MS suspension was added to resuspend the bacteria, followed by centrifugation; the supernatant was discarded. This was repeated three times. A 30 μL aliquot of acetosyringone was added. The ‘GL-3’apple leaves ‘were pre-cultured for 2 d and then transferred to the above *Agrobacterium* (28°C, 150 rpm for 30 min). After 2 d of dark culture, the apple leaves were transferred to apple leaf differentiation medium (MS + 0.5 mg/L NAA + 2.0 mg/L TDZ). The seedlings were excised from the leaf wound and inserted into the apple subculture medium to obtain transgenic apple seedlings. Transgenic plants were screened with kanamycin (kan). Three independent highly expressing lines were selected for the experiments. Transgenic apple calli were obtained by *Agrobacterium*-mediated transformation. Wild-type calli were incubated with *Agrobacterium* for 0.5 h, then transferred to selective medium for 30 d (An et al., 2020). The genetic transformation steps of tobacco leaves were the same as apple leaves. The gene CDSs are listed in **Supplementary Table 2**.

### 2.5 Measurements of relative electrical conductivity and malondialdehyde and chlorophyll contents

The appropriate amount of plant leaves were weighed, cut into small pieces, put in a centrifuge tube with ddH_2_O, soaked for 2 h at 24°C, and R1 conductivity was measured with the DDS-307A conductivity meter. Then, the leaf mixture was heated in a boiling water bath for 25-30 min, cooled to room temperature, and R2 final conductivity was determined. Relative electrical conductivity (%) = (R1/R2) × 100 (%). The thiobarbituric acid-based method was used to determine MDA content. Absorbance was determined at 450, 532, and 600 nm using a UV-vis spectrophotometer-2450 (Ma et al., 2017). Chlorophyll was extracted in 95% ethanol; the details are provided in An et al. (2021).

### 2.6 Yeast two-hybrid assays

The *MdNAC29-pGBKT7* vector was constructed and transferred into receptor cells and cultured on -Trp/X-α-gal medium at 30°C to verify autonomous activity. Screening of MdNAC29-*PGBKT7* vector yeast two-hybrid cDNA libraries: Denature 20 µL of Carrier DNA at 98°C for 5 min,4°C for 5 min, and repeat the process. Add 600 µL of Y2H Gold yeast receptor cells, 20 µL of pre-cooled MdNAC29-*PGBKT7* vector plasmid and 20 µL of apple cDNA library, 2.5 mL of PEG/LiAc to 15 mL centrifuge tubes under ice bath conditions. Mix lightly with a water bath at 30°C for 45 min (turn and mix every 15 min). Add 160 µL of DMSO to the centrifuge tube, turn and mix, then mix in a water bath at 42°C for 20 min (turn and mix every 5 min). Centrifuge at 5400 rpm for 5 min and discard the supernatant. Add 3 mL of YPDA liquid medium to the centrifuge tube, 30°C, 220 rpm for 90 min. Centrifuge at 5400 rpm for 5 min and discard supernatant. Add 1 mL of sterile 0.9% NaCl solution to the centrifuge tube and apply 100 µL of the suspension to SD/-T-L medium and incubate for 3-5d at 30°C. Select the monoclonal clones that turn blue and re-line them to SD/-Ade/-His/-Leu/-Trp/X-α-gal medium. Screened MdPP2-B10 (NCBI: XM_008361688.3) was inserted into the *pGADT7* vector following the Y2H assay steps as described previously (Li et al., 2021a).

### 2.7 Bimolecular fluorescence complementation assays

The *MdNAC29* CDS was cloned into the *pSPYCE-35S/pUC-SPYC*E vector to produce MdNAC29-YFP^C^. The full-length *MdPP2-B10* CDS was cloned into the *pSPYNE-35S/pUC-SPYNE* vector to produce MdPP2-B10-YFP^N^. The recombinant plasmids were transferred into *Agrobacterium* GV3101 and infected with onion epidermal cells using the same method as described in the Subcellular localization section.

### 2.8 Yeast one-hybrid assays

Full-length *MdNAC29* was inserted into the *pGADT7* vector, and the *MdDREB2A* promoter fragment (1264-1463bp upstream of ATG) was ligated into the *pAbAi* vector. The linearized plasmids were transferred into Y1H Gold yeast receptor cells and verified using Matchmaker one (Takara Bio, Shiga, Japan) to screen the bait strains for the AbA minimum inhibitory concentration. Different combinations of recombinant were co-transformed into Y1H yeast strains and cultured at -L-U+AbA_min_, observed, and photographed. (Li et al., 2017).

### 2.9 Dual-luc assays

The *MdDREB2A* promoter fragment was inserted into the *Luc* vector, and *MdNAC29* was inserted into the *pGreenII0029-62SK* vector. The different combinations of plasmids were transformed into *Agrobacterium* GV3101 strain, which were transiently injected into tobacco leaves, placed in the dark for 12 h, and incubated in a light incubator. The injected tobacco leaves were sprayed with D-luciferin sodium salt after 48-72 h and observed for fluorescence using a live imager (Xenogen, Alameda, CA, USA).

### 2.10 RNA sequencing

The RNA used for RNA-seq was extracted from Wild type (WT) and *MdNAC29* transgenic ‘Orin’ apple calli. Transcriptome sequencing included RNA extraction, detection, library construction, and sequencing. First, Pearson’s correlation coefficient (*R^2^*) analysis was used as an indicator to assess the correlations in the samples, with an *R^2^* value closer to 1 indicating that the two replicate samples were more correlated (**Supplementary Figure S1**). DEGseq2/edgeR was used to screen differentially expressed genes (DEGs) with q value < 0.05 and | log2 FoldChange | ≥ 1, and the total number of DEGs, the number of upregulated genes and the number of downregulated genes were counted. The sequencing data were filtered to obtain the clean data, which was compared with the apple genome to obtain the mapped data for structural analysis, such as variable splicing analysis, novel gene mining and gene structure optimization, differential expression analysis, KEGG functional annotation of differentially expressed genes, and functional enrichment based on the expression of genes in the samples or groups (Wang et al., 2010; Pertea et al., 2015; Yi et al., 2016; Chen et al., 2018).

### 2.11 Statistical analyses

Three biological repetitions were performed for each treatment, and each biological repetition had three technical replicates. Different letters represent significant differences (one-way ANOVA was performed using SPSS; SPSS Inc., Chicago, IL, USA, Tukey-Kramer test, *p* < 0.05). The data analysis was carried out using Student’s *t*-test, and asterisks denote significant differences. GraphPad Prism 6 software (GraphPad Software Inc., La Jolla, CA, USA) was used for drawing.

## 3 Results

### 3.1 *MdNAC29* is induced by drought stress and localized in the nucleus

**Figure 1:**
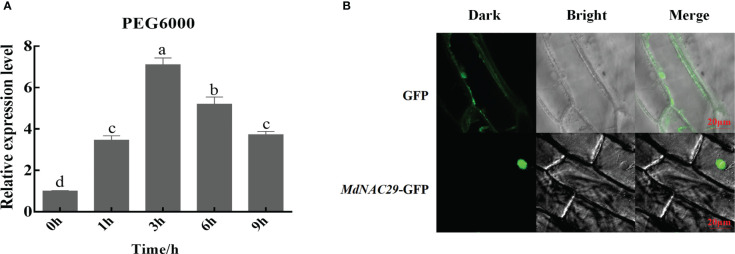
Effects of drought stress on the *MdNAC29* transcript level and subcellular localization analysis. ‘GL-3’ apple seedlings grown at room temperature were treated with 18% PEG6000. **(A)** The transcript level of *MdNAC29* was determined using qRT-PCR. **(B)** Subcellular localization of *MdNAC29* in onion epidermal cells. Different letters represent significant differences (one-way ANOVA was performed using SPSS; SPSS Inc., Chicago, IL, USA, Tukey-Kramer test, p < 0.05).

NAC29 gene expression is induced by multiple abiotic stressors in plants (Huang et al., 2015; Wang et al., 2015; Seok et al., 2017). To further investigate the response of *MdNAC29* to drought stress, we subjected ‘GL-3’ apple seedlings to 18% PEG6000 treatment, as shown in **Figure 1A**. The *MdNAC29* transcript level increased under the PEG treatment to reach its highest level at 3h followed by a decrease, indicating that *MdNAC29* may play an important role in the drought stress response. The subcellular localization experiments revealed that *MdNAC29* was localized in the nucleus (**Figure 1B**).

### 3.2 *MdNAC29* negatively regulates drought tolerance

**Figure 2:**
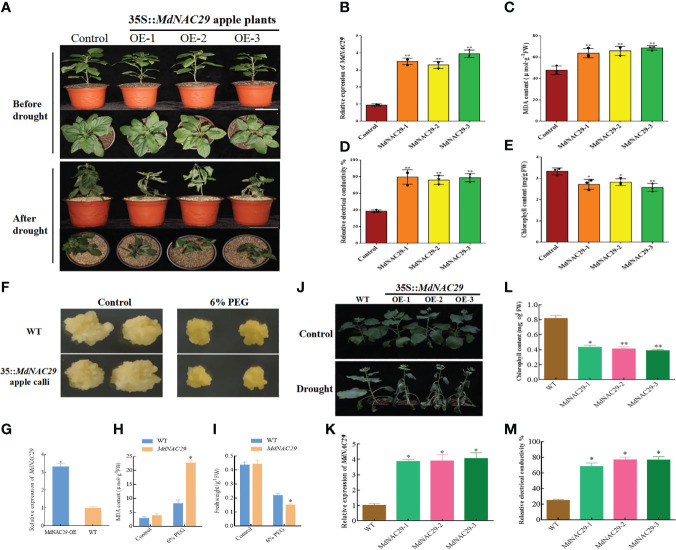
*MdNAC29* reduces drought resistance in apple plants, calli and tobacco. **(A)** Phenotypes of control and *MdNAC29* transgenic apple plants after 25d of drought treatment. Scale bar: 10 cm. **(B)** The transcript level of *MdNAC29* was determined using qRT-PCR in transgenic apple. **(C–E)** MDA, relative electrical conductivity and chlorophyll content of control and *MdNAC29* transgenic apple after drought treatment. **(F)** Phenotypes of WT and *MdNAC29* transgenic apple calli after 15d of 6% PEG. **(G)** The transcript level of *MdNAC29* was determined using qRT-PCR in transgenic apple calli. **(H, I)** MDA, fresh weight of WT and *MdNAC29* transgenic apple calli after 15d of 6% PEG. **(J)** Phenotypes of WT and *MdNAC29* transgenic tobacco after 10d of drought treatment. **(K)** The transcript level of *MdNAC29* was determined using qRT-PCR in transgenic tobacco. **(L, M)** Chlorophyll content and relative electrical conductivity of WT and *MdNAC29* transgenic tobacco after drought treatment. “*” denote significant differences p < 0.05, “**”denote significant differences p < 0.01.

We further validated the biological functions of *MdNAC29* by constructing the overexpression vector (35S::*MdNAC29*) and obtaining the transgenic materials. First, control and *MdNAC29-* over-expressing apple plants were exposed to drought conditions for 25 d. More leaf wilt symptoms were observed in response to drought stress in *MdNAC29* transgenic apple plants than in the control (**Figure 2A**). The qRT-PCR was used to detect the expression level of *MdNAC29*, and *MdNAC29* expression was significantly upregulated in the overexpressing *MdNAC29* transgenic apple plants (**Figure 2B**). To assess the drought tolerance of *MdNAC29* transgenic apple plants, we measured relative electrical conductivity as well as malondialdehyde (MDA) and chlorophyll contents and found that the overexpressing *MdNAC29* transgenic apple plants had higher MDA content (**Figure 2C**), higher relative electrical conductivity (**Figure 2D**), lower chlorophyll content (**Figure 2E**) than the control. These results suggest that overexpressing *MdNAC29* reduced drought tolerance in apples. Next, 7-day-old WT and transgenic (35::*MdNAC29*) apple calli were transferred to MS medium supplemented with 6% PEG and cultured for 15 d. As shown in **Figure 2F**, overexpressing *MdNAC29* apple calli resulted in more pronounced drought-sensitive phenotype, with lower fresh weight and higher MDA content compared to the WT (**Figures 2G–I**). In addition, consistent with the results of the transgenic apple plants, overexpressing *MdNAC29* in tobacco (**Figures 2J, K**) by heterologous transformation resulted in lower chlorophyll content (**Figure 2L**) and higher relative electrical conductivity (**Figure 2M**) compared to the WT. Taken together, these results suggest that *MdNAC29* acts as a negative regulator in drought tolerance.

### 3.3 Transcriptome analysis of overexpressing *MdNAC29* in apple calli

#### 3.3.1 Differential gene analysis

**Figure 3:**
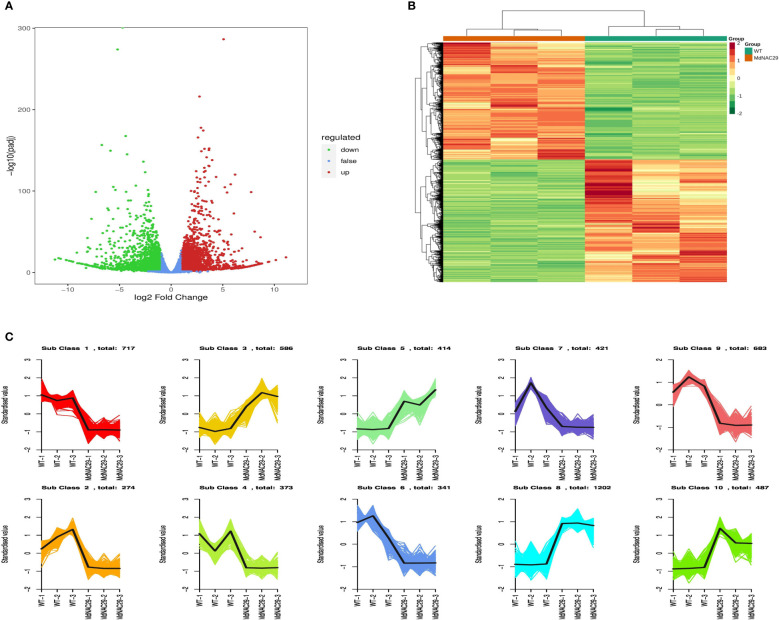
Screening and classification of differentially expressed genes between WT and *MdNAC29* transgenic apple calli. **(A)** Scatterplot of differentially expressed RNAs. **(B)** Heat map of clustering of differentially expressed genes. **(C)** Kmeans analysis plot of differentially expressed genes.

To further investigate the regulatory pathways associated with *MdNAC29* drought tolerance, we performed RNA-seq analysis. Compared with the control (WT), 5,498 DEGs were detected, of which 2,689 genes were upregulated and 2,809 genes were downregulated (**Figure 3A**). The DEGs in the groups (*MdNAC29* and WT) were taken and set together as differential gene sets for hierarchical cluster analysis, and the data were normalized using the Z-score. A cluster heat map was plotted for each grouping (**Figure 3B**). The FPKMs of all differential gene concatenations were normalized using R language to plot the Kmeans cluster analysis. The same class of genes had similar trends under the different treatments and may have similar functions (**Figure 3C**).

#### 3.3.2 GO classification and KEGG annotation of the DEGs

**Figure 4:**
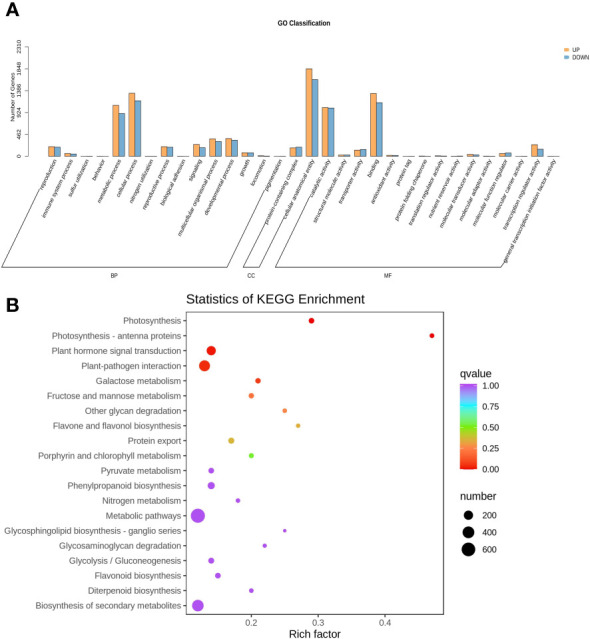
GO classification and KEGG annotation analyses of differentially expressed genes between the WT and *MdNAC29* transgenic apple calli. **(A)** GO category histogram. **(B)** KEGG enrichment scatterplot.

The GO classification analysis revealed that overexpressing *MdNAC29* significantly enriched the DEGs in the biological processes of cellular composition and molecular functions (**Figure 4A**). We performed KEGG annotation and GO classification analyses of the DEGs generated by overexpressing *MdNAC29* to further identify the functions of the genes. The KEGG enrichment analysis showed that the DEGs produced by overexpressing *MdNAC29* were mainly enriched in the pathways of photosynthesis, plant hormone signal transduction, plant-pathogen interaction, and porphyrin and chlorophyll metabolism compared to the WT (**Figure 4B**). We selected the 50 GO-Terms with the lowest q-values in the GO enrichment analysis results and found that 11 genes regulated leaf senescence and 17 genes were related to chlorophyll binding (**Supplementary Figure S2**), and these biological processes and molecular functions were closely related to drought tolerance.

### 3.4 Overexpression of *MdNAC29* represses the expression of drought tolerance genes

**Figure 5:**
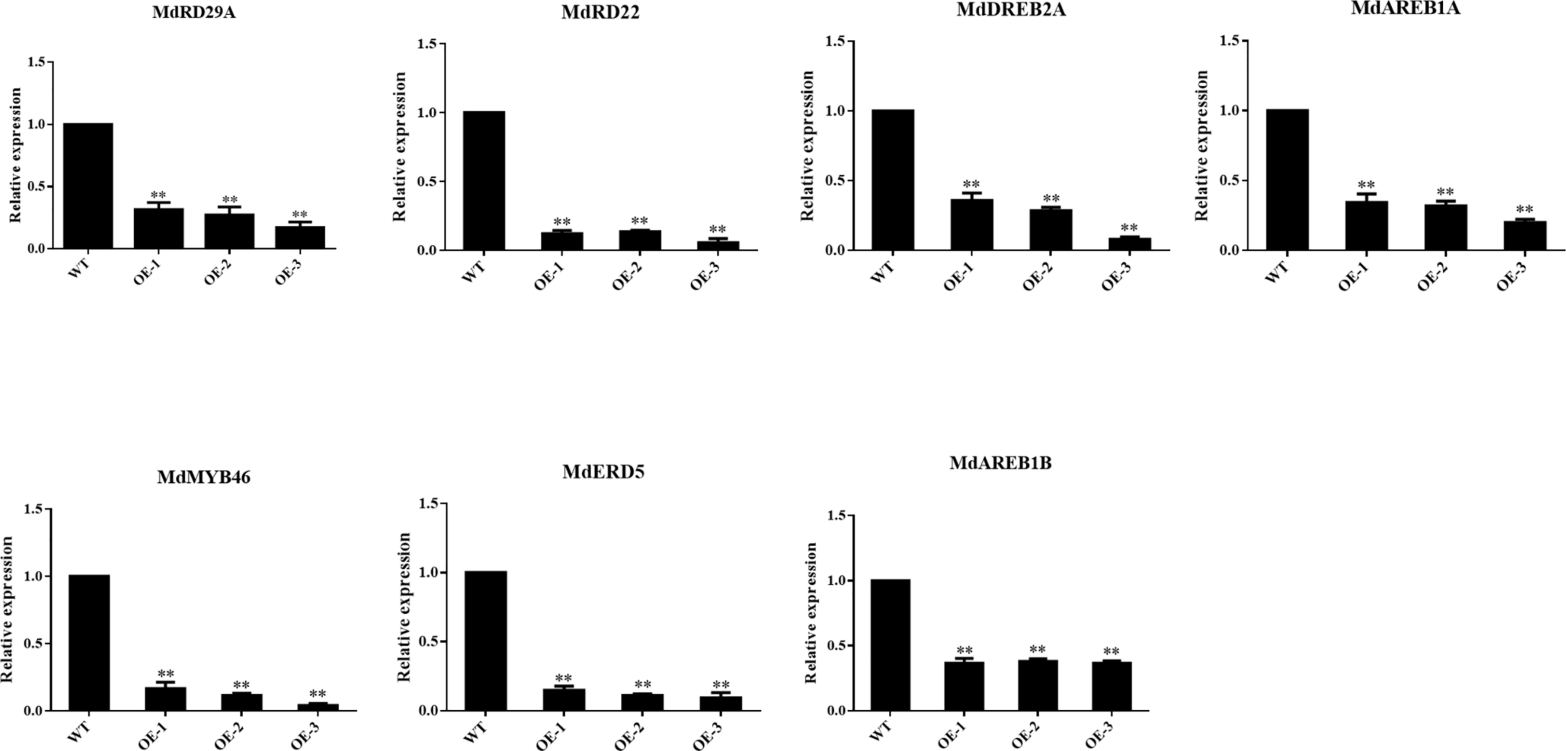
Expression analysis of key genes associated with drought tolerance in *MdNAC29* transgenic apple plants. *MdERD5*: MD09G1080200, *MdRD22*: MD15G1098800, *MdRD29A*: MD01G1201000, *MdAREB1B*: MD05G1082000, *MdAREB1A*: MD15G1081800, *MdDREB2A*: MD01G1158600, *MdMYB46*: MD03G1176000. “*” denote significant differences p < 0.05, “**”denote significant differences p < 0.01.

To further analyze the effect of overexpressing *MdNAC29* on genes associated with drought stress, we selected the key genes associated with drought stress and analyzed their expression levels in *MdNAC29* transgenic apple plants by qRT-PCR (**Supplementary Table 3**). As shown in **Figure 5**, overexpressing *MdNAC29* in apple plants resulted in a significant decrease in the expression of genes (*MdERD5, MdRD22, MdRD29A, MdAREB1, MdDREB2A*, *MdMYB46*) that positively regulate drought tolerance (Chen et al., 2019; Lian et al., 2021; Li et al., 2022; Liu et al., 2022), further indicating that *MdNAC29* negatively regulates drought tolerance.

### 3.5 MdNAC29 binds directly to the *MdDREB2A* promoter to repress its expression

**Figure 6:**
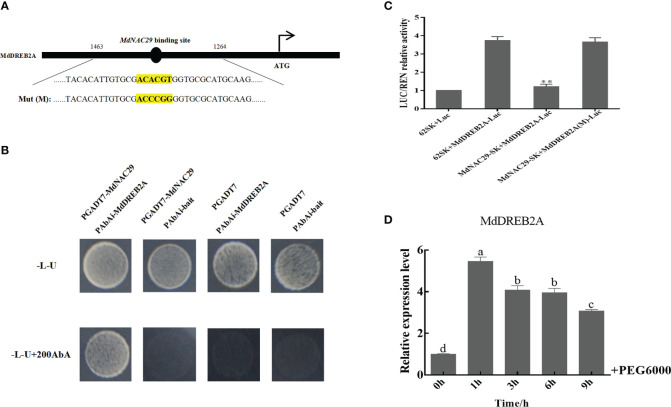
MdNAC29 repressed expression of the drought tolerance gene *MdDREB2A*. **(A)** Structural analysis of *MdDREB2A*. **(B)** YIH assays of MdNAC29 bound to the *MdDREB2A* promoter *in vitro*. **(C)** LUC/REN relative activity was detected from the effects of MdNAC29-*62SK* on the expression of *MdDREB2A-Luc*. **(D)** Expression pattern of *MdDREB2A* in response to PEG treatment. Different letters represent significant differences (one-way ANOVA was performed using SPSS; SPSS Inc., Chicago, IL, USA, Tukey-Kramer test, p < 0.05). **”denote significant differences p < 0.01.

The NAC TFs that bind to the DREB2A promoter to regulate drought tolerance in plants have been widely studied (Jin et al., 2017; Lian et al., 2021). We speculate that MdNAC29 in apple may also bind to the *MdDREB2A* promoter to regulate drought tolerance. We analyzed the *MdDREB2A* promoter sequence (**Supplementary Table 4**) and found that it contained the NAC TF binding site (ACACGT) (**Figure 6A**). Y1H assays were performed to test whether MdNAC29 directly binds to the *MdDREB2A* promoter. Only the co-transformed with *PAbAi-MdDREB2A* and *PGADT7*-MdNAC29 grew normally on -L-U+200AbA deficient medium (**Figure 6B**), indicating that MdNAC29 directly binds to the *MdDREB2A* promoter. To further verify how MdNAC29 regulates the expression of *MdDREB2A*, we performed the dual luciferase reporter assay. The LUC/REN relative activity of the co-expressed MdNAC29-*62SK*+*MdDREB2A-Luc* combination decreased significantly compared to the combination of *62SK*+*MdDREB2A-Luc* (**Figure 6C**), the mutation of the NAC binding site (ACACGT) in the *MdDREB2A* promoter (ACCCGG), MdNAC29 did not affect *MdDREB2A* expression, indicating that MdNAC29 inhibited *MdDREB2A* expression. In addition, qRT-PCR analysis revealed that *MdDREB2A* expression reached its highest level after 1 h of PEG treatment and then gradually decreased (**Figure 6D**).

### 3.6 MdNAC29 interacts with MdPP2-B10 in response to drought stress

**Figure 7:**
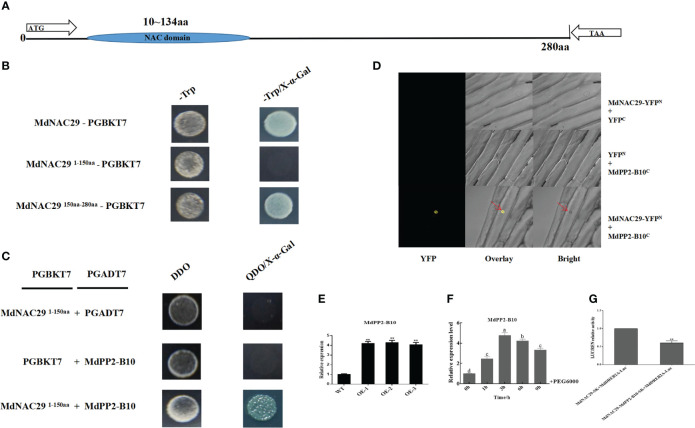
MdNAC29 directly interacts with MdPP2-B10 and MdPP2-B10 responds to PEG treatment. **(A)** Structural analysis of MdNAC29. **(B)** The MdNAC29 protein sequence (1-150 aa) fragment had no autonomous activation activity. **(C)** Y2H assays verified the interaction between MdNAC29 and MdPP2-B10. The *PGADT7* and *PGBKT7* empty vectors were used as negative controls, DDO:-T-L medium, QDO:-T-L-A-H medium. **(D)** BiFC assays to verify the interaction between MdNAC29 and MdPP2-B10. **(E)** Expression analysis of MdPP2-B10 in *MdNAC29* transgenic apple plants. **(F)** Expression pattern of MdPP2-B10 in response to PEG treatment. **(G)** LUC/REN assay to analyze the effect of MdPP2-B10 on MdNAC29-mediated transcriptional activation of *MdDREB2A*. Different letters represent significant differences (one-way ANOVA was performed using SPSS; SPSS Inc., Chicago, IL, USA, Tukey-Kramer test, p < 0.05). **”denote significant differences p < 0.01.

To further analyze whether MdNAC29 interacts with MdPP2-B10 to co-regulate drought tolerance, we analyzed the MdNAC29 structure and determined that the NAC domain was located at 10-134 aa (**Figure 7A**). MdNAC29 was tested for autonomous activity and had strong autonomous activation activity (**Figure 7B**). We verified by segmentation (1-150aa and 150-280 aa) and found no autonomous activation at 1-150 aa, which could be used in subsequent Y2H assays. After preliminary screening by the Y2H assay, only the combination of MdNAC29^1-150aa^-*pGADT7* + MdPP2-B10-*pGBKT7* grew normally on QDO/X-α-Gal medium, indicating that MdNAC29 interacts with MdPP2-B10 (**Figure 7C**), and the interaction between MdNAC29 with MdPP2-B10 was further confirmed by BiFC assay (**Figure 7D**). The Y2H and BiFC primers are listed in **Supplementary Table 1**. Furthermore, the expression of MdPP2-B10 was significantly higher in overexpressing *MdNAC29* apple plants compared to WT (**Figure 7E**). and we analyzed the MdPP2-B10 expression pattern after the PEG6000 treatment and found that MdPP2-B10 responded to drought stress (**Figure 7F**). To verify whether MdPP2-B10 had an effect on MdNAC29-mediated transcription of *MdDREB2A*, it was further analyzed by LUC/REN relative activity test, which showed that MdPP2-B10 enhanced the repressive effect of MdNAC29 on the transcriptional activity of the downstream target gene *MdDREB2A* (**Figure 7G**).

## 4 Discussion

Plants have evolved morphological, physiological, and molecular resistance mechanisms in response to abiotic stress, and among these molecular mechanisms are the regulation of gene expression by TFs and the roles of some functional genes. NAC genes are TFs specific to plants that affect plant growth and development while also playing a role in transcriptional regulation of various abiotic stressors, such as drought, salt, and cold stress as well as pathogen infection (Sun et al., 2012; Tak et al., 2017). Members of the NAC gene family are differentially expressed in response to abiotic stress. Here, we isolated the NAC TF *MdNAC29* in apples. Our results show that *MdNAC29* was localized to the nucleus and responded to drought stress (**Figure 1**). Overexpressing *MdNAC29* negatively regulated drought tolerance in transgenic apple plants, calli, and tobacco (**Figure 2**). *MdERD5, MdRD22, MdRD29A, MdAREB1, MdDREB2A* and *MdMYB46* have been widely reported to enhance drought tolerance in plants (An et al., 2018; Chen et al., 2019; Li et al., 2022; Liu et al., 2022). We speculate that *MdNAC29* regulates the expression of these drought-responsive genes. The qRT-PCR results showed that overexpressing *MdNAC29* in apple plants significantly downregulated the expression of these genes (**Figure 5**).

The DREB TF family is essential in improving stress-resistant plant varieties (Zhao et al., 2012; Yoshida et al., 2014). The DREB transcription factor (*TaDTG6-B*) ameliorates drought tolerance in wheat (Mei et al., 2022). *MdDREB2A* is a key drought-tolerance gene in apples (Chen et al., 2020; Li et al., 2022). In our study, we analyzed the *MdDREB2A* promoter sequence and detected a NAC binding site (ACACGT), which further confirmed that MdNAC29 could bind the *MdDREB2A* promoter and inhibit its expression (**Figure 6**). The RNA-seq results showed that overexpressing *MdNAC29* positively regulated leaf senescence (**Supplementary Figure S2**). NAC29 also positively regulates leaf senescence in *Arabidopsis* and tobacco (Liang et al., 2014; Ma et al., 2020), suggesting that the NAC29 gene is highly conserved across species. Hence, our study provides a new basis for future analysis of *MdNAC29* regulation of leaf senescence. Furthermore, DREB2A may bind to the DRE element (A/GCCGAC) of target gene promoters (e.g., COR, RD, LTI, and DHN) to regulate their expression during tolerance to stress (Liu et al., 2014). Interestingly, the DRE motifs were present on the *MdAREB1A* and *MdRD22* promoters (**Supplementary Table 5**). We hypothesized that *MdDREB2A* regulates the expression of *MdAREB1A* and *MdRD22* to affect drought tolerance in apples.

F-box proteins are widely found in eukaryotes and are important components of the SCF-E3 ubiquitin ligase complex. These proteins are involved in important physiological processes, such as cell cycle regulation, gene transcriptional regulation, apoptosis, and signal transduction, through the ubiquitin-proteasome pathway (Nakayama and Nakayama, 2006). In *Arabidopsis*, BAF1 (F-box E3 ubiquitin ligase BES1-ASSOCIATED F-BOX1) mediates the degradation of the BES1 (Brassinosteroid-activated transcription factor) through selective autophagy (Wang et al., 2021). F-box proteins respond to a wide range of abiotic stressors. In *Arabidopsis*, the F-box protein DOR specifically interacts with ASK14 and CUL1 to negatively regulate drought tolerance. DOR inhibits ABA-induced stomatal closure under drought stress (Zhang et al., 2008). Overexpression of the grapevine F-box protein VvF-box5 significantly increases the survival rate of transgenic *Arabidopsis* under drought treatment (Yu et al., 2018). Our study revealed that MdNAC29 interacted with MdPP2-B10, the expression of MdPP2-B10 was significantly higher in overexpressing *MdNAC29* apple plants compared to WT (**Figure 5**), and MdPP2-B10 responds to drought stress (**Figure 7E**). Moreover, LUC/REN assays showed that MdPP2-B10 enhanced the repressive effect of MdNAC29 on the transcriptional activity of the *MdDREB2A* (**Figure 7F**). Most of the reported F-box proteins had been shown to function via SCF-dependent protein degradation, some F-box proteins that do not depend on the formation of the SCF complex to perform their functions, but rather alter the expression of downstream genes by regulating transcription factor activity, thereby affecting the plant’s resistance response. (Jia et al., 2018). We speculate that MdPP2-B10 may modify the MdNAC29 protein and affecting its expression without causing its degradation. Therefore, investigating whether MdPP2-B10 forms the SCF complex and the molecular mechanism of its interaction will be the focus of our future research.

**Figure 8:**
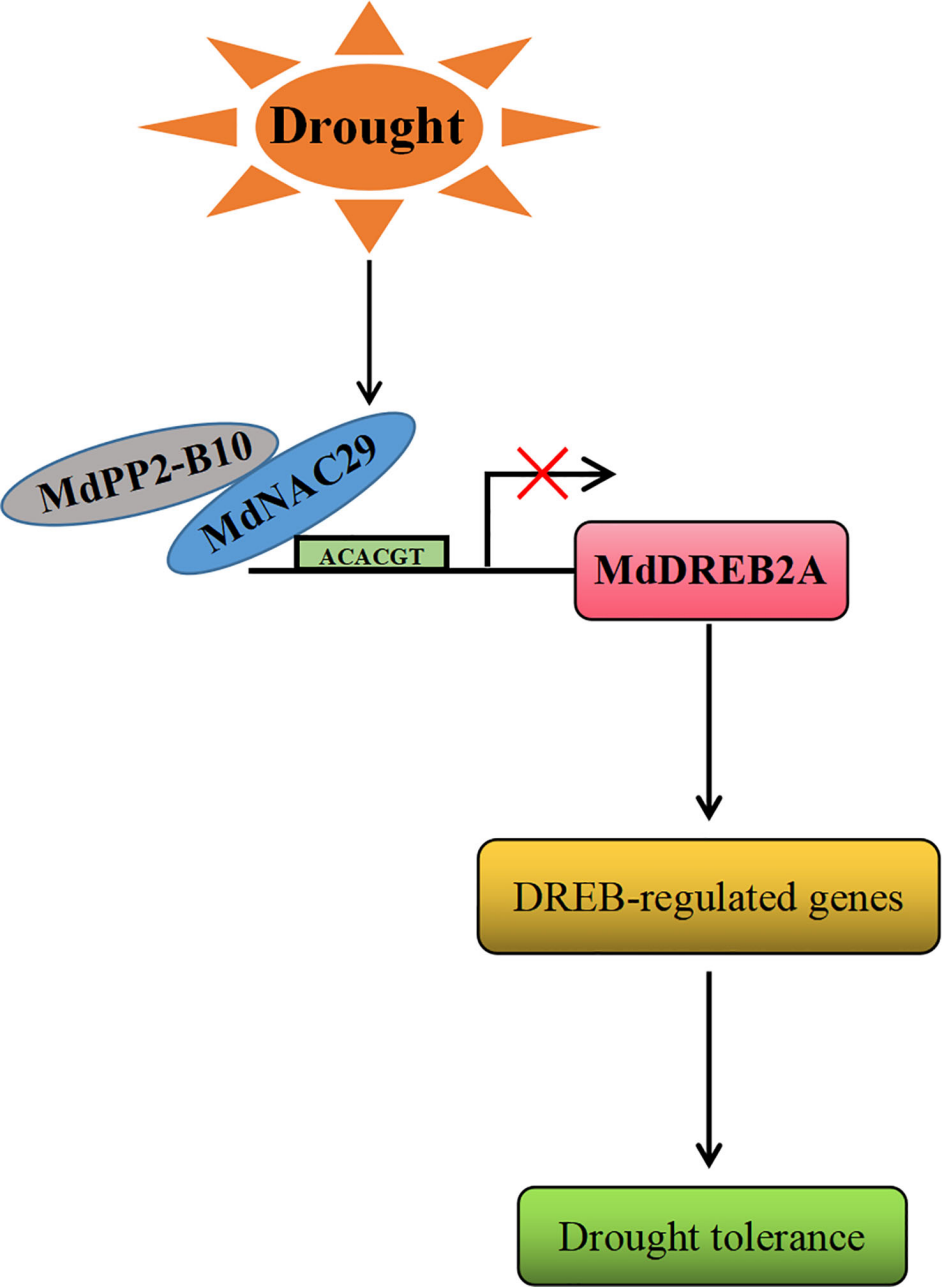
A working model for the regulation of drought tolerance by *MdNAC29*. Drought stress induced the expression of *MdNAC29*, which was repressed to negatively regulate drought tolerance by binding to the *MdDREB2A* promoter element (ACACGT). Drought stress induced MdPP2-B10 expression, which interacted with MdNAC29 to jointly regulate drought tolerance.

In conclusion, based on the results of this study, we propose a working model for the regulation of drought tolerance by *MdNAC29* in response to drought stress (**Figure 8**). Drought stress induced the expression of *MdNAC29*, which repressed its expression by binding to the *MdDREB2A* promoter motif (ACACGT), which, in turn, affected the expression of its downstream target genes to negatively regulate drought tolerance. Drought stress induced the expression of MdPP2-B10, which interacted with MdNAC29 to co-regulate drought tolerance. Our findings provide new insight into the response mechanism of *MdNAC29* to drought stress.

## Data availability statement

The data presented in the study are deposited in the NCBI Sequence Read Archive (SRA) repository, accession number PRJNA946317.

## Author contributions

LL, WX, SL, and XJ designed the study. SL and XJ performed the experiments. SL, XJ, QT, and BW analyzed the data. SL and XJ wrote the paper. XF, DL, XC, WX, and LL participated in the manuscript amending. SL and XJ are the co-first author and contributed equally to this work. LL and WX are the co-corresponding author. All authors contributed to the article and approved the submitted version.
