# Supplementary figures and images for: The NAC transcription factor MdNAC29 negatively regulates drought tolerance in apple
Source: Front Plant Sci. 2023 Jul 6;14:1173107. doi: 10.3389/fpls.2023.1173107 (PMC10359905; doi:10.3389/fpls.2023.1173107)

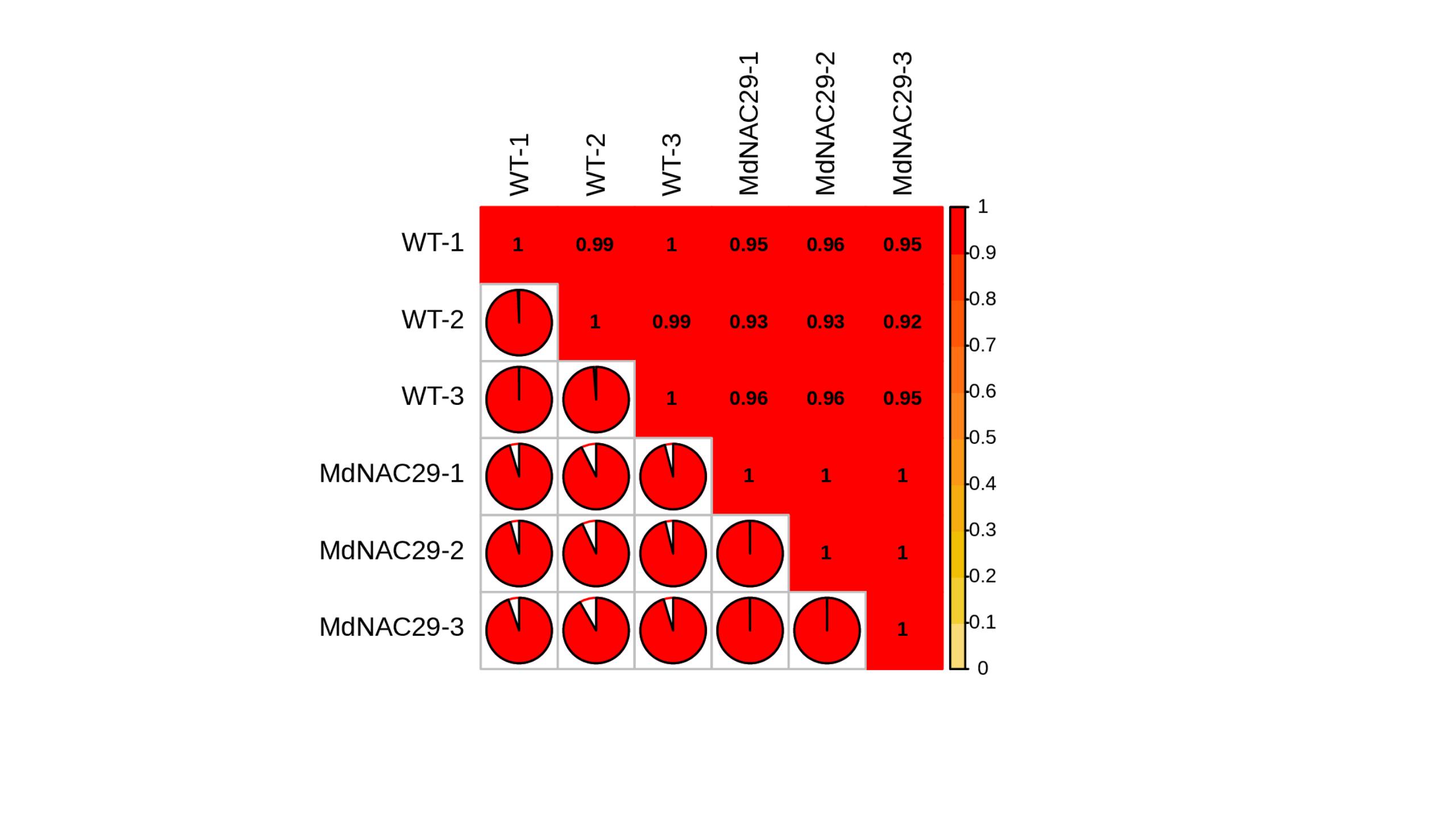

Supplement: Supplementary file 1 [file Image_1.jpeg]

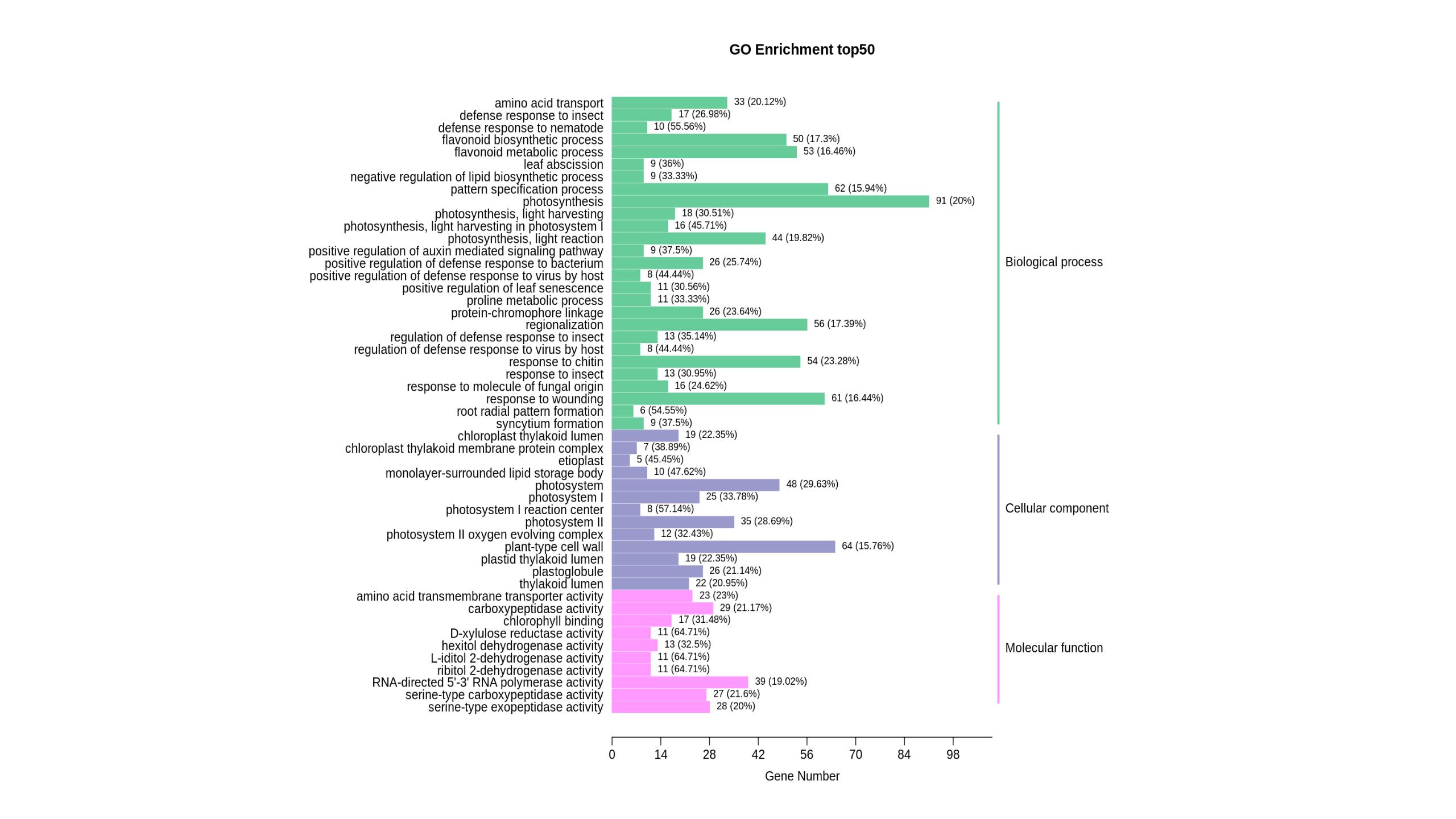

Supplement: Supplementary file 2 [file Image_2.jpeg]
