# Supplementary material for: The NAC transcription factor MdNAC29 negatively regulates drought tolerance in apple
Source: Front Plant Sci. 2023 Jul 6;14:1173107. doi: 10.3389/fpls.2023.1173107 (PMC10359905; doi:10.3389/fpls.2023.1173107)
Supplement: Supplementary file 4 [file Table_2.docx]

**MdNAC29-CDS**

ATGGAGGCAAAACGAAGCTCTGACCTTCCCCCTGGTTTTAGATTCCACCCAACTGATGAGGAACTCATCGTTTTTTACCTAAAAAACCAAGCCTCCTCAAGGCCCTGCCCTGTGTCCATCATCCCAGAGGTTGATATTTACAAGTTTGACCCGTGGGAGTTGCCTGAGAAGGCAGAGTTTGGAGAAAACGAATGGTACTTCTTCACCCCGAGAGACCGAAAGTACCCGAACGGGGTACGCCCGAATCGGGCAACCGTATCGGGTTACTGGAAAGCCACGGGAACAGACAAGGCAATTTACAGCGAGTCCAAGTACGTGGGGGTGAAGAAAGCTCTCGTGTTTTACCAGGGCAGGCCGCCAAAGGGGGTCAAGTCGGACTGGATTATGCACGAGTATCGCTTAAGCGACTCGCGAAAACAACAACCCAACAAGCACCTCGGGTCCATGAGACTGGATGATTGGGTCCTCTGCAGGATCTACAAAAAAAAGCATCCGGGCAAAGCTTATCTGGATCAAAAAGTGGAAGAAGATCAAAAAATTGAGATGAGAACACCAGAAACGGCGAAAGCCAATGAGGAACAAGTGATGTTGAAATTTCCACGGGCCTGTTCGATAACTAGCTTACTGGACATGGACTACTTGGGCCCGATCTCCCAACTTTTTAGTGACAATATTTCTGGATACGATTTTCAGACCAGCATGGCCGGCGCAGGAGCCGGCCAGGCTCAAATGTTTCAGTTCGGTGAAGTGCCGAATTACCAAAACACGACGGACTCCGGAAAATTCCAAGTGACGTCGGCTCAGACTAGCGTTTTTAACCACCAACCGTGGTTTGGACCGTAA

**MdNAC29 interacting genes - CDS**

**1、NCBI Sequence ID: XM_008349773.3**

**PREDICTED: Malus domestica glutaredoxin-C5-like (LOC103411116), mRNA**

atgcactacc agaccgagtc atggggctcc tgcatgccca caagaacctc catgggcata gaggaccctc tggagcgcat agagcggatg gcgtcggaga gcgcggtggt gatattcagt ataagcagct gctgcatgtg ccacgccatc aagcgcctct tctgcggcat gggcgtcaac cccactgtgt acgagctgga cgaggacccc agaggtaagg acttggagag ggcgttgatg aggctgctgg ggacctcctc cgccgtcccc gtcgtcttca tcggtggcaa actcgtcggc gcaatggaca gagtcatggc ctctcatatc aacggcactc ttgttcctct tctcaaagag gccggggctc tctggctctg a

**2、Sequence ID: XM_029106484.1**

**PREDICTED: Malus domestica BES1/BZR1 homolog protein 4-like (LOC114826390), mRNA**

atggcggcaa agactcaggc gttgatgagg ttgttgggga cctcatttgc cgttcccgtt gtcttcatcg atggcaagct cgctggcgca atggacaaag tcatggcctc tcatatcaac ggcaccactt accgcgaggg atgcaagccg gtcgaatata tggacgtgat gggtggatcc acattagcta gcccaagctc gtctttcctc ccaagtaatt atgcttccta caatccaagc caggctctta ctccttccct agcccaacat catcttccta tggatctcct gaacgctaat ggcagatccc ttatcccatg gctcaaaaat ctctcatatg catccttttc agcctcctca tccaaactac caaacctcta catccatggt ggctctatca gtgcttctgt tacccctcca tttagctccc caattgccaa gacaccgaga attagaattg actgggatga ccagtgtgct ccgccaggtt gggctaggta g

**3、Sequence ID: XM_008392899.3**

**PREDICTED: Malus domestica ferredoxin, root R-B1 (LOC103453352), mRNA**

atgtcagc catggcaacc gttcaagttc ccacccaatg catgttcaga catgcgcccc agaaacagtt caccagttca

attgtgaagc tatcttctcc ccttggatct gtgaagaata tctccaagtc ctttggcttg aaagcatcct caaacttccg ggcatcaatg gaggtataca aggtgaagct gattgggcca gatggtgtgg agaatgagtt tgaggcggcg gatgattgct acatcttaga tgcagctgag aatgctggag ttgaactgcc atattcttgc agggctgggg catgctctac ctgtgcagggaagctggcatcgggttcagt ggatcagtcc gatggttcat tcctcgacga caaccaaatgaaggagggtt atgtgctgac ttgtgtatcg tacccaactt cagaccttgt gattcacact cacaaggaga gtgagctgta ttag

**4、Sequence ID: XR_527643.3**

**PREDICTED: Malus domestica uncharacterized LOC103423606 (LOC103423606),**

tttggacatc acaaaaaaag ccctaaggac aaaaccggca taaaaaatag ttttaaaatt agggtttcat ctcgtatata gcctccttgc ctcgacctcc aactccgact ttcagagaca actcgcagcc tccagcaaga aaccatcaaa atgccgaagc agatccatga gattaaggat ttccttctaa ccgcaagaag gaaggatgcc cgcaatgtca aaatcaagag gaccaaggat gctgttaagt tcaaggttcg gtgctccaag tacctttaca cactttgtgt gtttgattcc gacaaggcca acaagttgaa gcaatctctt cctccaggtt tgaacgttca ggatccttga accaggattt tggagtggct tgtcggcaca ttctagtgaa tgtagcaatg gaatgaaatt tgtactttga aagattttta tggccggatg atgttggtgt tagatgttag aactccgaga tgtttgtggt taatgtgttt tgacttgagt taattgttta gatgttttgg ttatggatcc atcgggtttg tgtttgcttg a

**5、Sequence ID: XM_008390541.3**

**PREDICTED: Malus domestica F-box protein At2g02240-like (LOC103451108),**

atggatc tgcaggcctt gccggaaggc tgcatagcca ccgttatctcgctgaccacg cctcgagacg cggccaccat gtcctcggtt tcgaggagtt tcaggtcggc agcggaatcc ggcgccgtct ggggcaggtt ccttcccccc gatctccaca ctatcctgtc ctcctcgtca ccctccatgt ccgtacatcc gccgcatgtg gctccctccg cttccaaaac caagaaggag ctttacctcg ctctgtgcga caacccagtt ctcgtcgaac agggcaagtt gggcttttca ctagacaaat ggagtgggaa gaaatgctat atgattgctg caagggccct ttcaatcgtt tgggccgaca ctcctcagta ctggaaatgg atttctatcc ccgactcaag gtttgaggag gtggcagagc ttgtggatgt gtgttggctt gaaatccatg gcaaaattgagactcggatg ctgtccccat ccacccttta caaagcttat cttgtattca agacaactgcagaggcttct ggacttgagc tccgcgatgt ggaggtcaca gtgggtttaa ttggggagat

cactgaggcc caagaccaga ccagggagat caatgaggtc cagtatccga aagagcgcaacgacgggtgg ctggagatag agatggggga gttcttctgt gaagagggtg aaggtggggagttgaagatg agttgcttgg agacaaatgg tcactggaag agcggcctca ttgttcaagg gattgaagtc aggcccgaaa ggatgtag

**6、Sequence: XM_008361688.3-MdPP2-B10**

**PREDICTED: Malus domestica F-box protein PP2-B10-like (LOC103423598),**

atgtct aaacaaatta caagaaccag aagaaaacac atggatctgc aggccttgcc ggaagggtgc atagccaccg ttatctcgct gaccacgcct cgagacgcgg gcacgttgtc ctcggtttcg tggagtttca ggtcggcagc ggattccgac gccgtctggg gcaggttcct tccccccgat atccacacta tcctgtcatc ctcgtcaccc tccatgtccg tacagccgcc gcacgtgggtccctcctccg cttcaaaaac gaagaaggag ctttacctcg ctctgtgcga caacccagtt ctcatcgaac agggcaagtt gagcttttca ctggacaaat ggagtgggaa gaaatgctat atgattgctg caagggccct ttccatcgtt tgggccgaca ctcctcagta ctggaaatgg atttctatcc ccgactcaag gtttgaggag gtggcagagc ttgtggatgt gtgttggctt gaaatccatg gcagaattga gacacggatg ctgtccccat ccacccttta caaagcttat cttgtattca agacaactgc acaggcttat ggatttgagc accgcgctgc ggaggtcaca gtgggtttaa ttggggagca gcgcacgaat caaaatgtgt ttctgggcgc tcggagagtccagacccgag gccagggcta tcacatggcc aggcctcggg gtattcgccg aacatacatt ctagaccagc ccagggagat caatgaggcc cagtacccga aagagcgcaa cgacggatggctggagatag agatggggga gttcttttgt gaaggaggtg aaggtgggga gttggagatg agttgcttgg aaacaaaggg tagacactgg aagagcggcc tcattgttca agggattgag atcagaccca aaaggatgta a
