# Supplementary material for: The NAC transcription factor MdNAC29 negatively regulates drought tolerance in apple
Source: Front Plant Sci. 2023 Jul 6;14:1173107. doi: 10.3389/fpls.2023.1173107 (PMC10359905; doi:10.3389/fpls.2023.1173107)
Supplement: Supplementary file 6 [file Table_4.docx]

**The promoter sequence of MdDREB2A**

CCTTCAAACTTTGTCTAGCCGCTGCGTCAGCAAACACGCAAGCTGCCACCTCATCACTTTGTTAAACTCCAGCGCACACCATGGATTTGGATCCGGAATAGAATACTTTTTGTAAGAATCCAGATGATTAATCAATTGTATTCATTCACCGTGCGGTTAGAAATTATTTTTAATTTTTAATTTAAAATTGAATATAAATAGTACCTAGCGAAAATTGACCGCATGATGTACGATGAATGGACATGATTGATTGATCTTCAGATCCCCATAAAGAGGATCCAGACAGGATCCTTGTCTTTTGGTTCCCATTCGGATTCAAATTGTGGGGTTCTAGGGATCTTCACAGCTTAGTCATTCATCGTGCATCGTGTGGTCAGAAATCATTTGATTTTTTTATTTAAAATTAAACATAAATAATACCTGACGAAAATTAACCACAAATGAAAAGTTAAAGTATAAAGATCTCTAAGATCGTCACAAAGTAAATCTGGAGAATATCCTCTTTCCGCACACCACGACCCAATCGCCTTCAGTGTT**CAATTCTCCACCACACGGAATTGAACATGTCACTCATGACATGATTAAGTTAGGCGTCCAAGTGAACTAAAATTGCCACGTTGCCCTACACATTGTGCGACACGTGGTGCGCATGCAAGAAGTCGACGAGGAAGACAGGATAACATAATACTGTCCGAACGAGGGAGACATGCTGTTTTCCCTTGGTAGTGAAAAATGGT**TCGTGGTTGAGCCCATGGGGTTGAGAAGAATTACGATGCAATTAGCACCTCAATGCCCCGTGTTCGACGTTAATCATGCAAAAACAATTTAACGTGTGAAATGTCGAAAATAAAAAAATTTCAAGATGGTAAATTGAGTATCCTAGATAGACATTCATATAAAAATGCGATATAAAGAGAGAGCAAAAGATGAAGAACTTACCCTTCGTAGAATTTCAAAAAATAAATTGATCGTGATTCATATGTTTTTATCCCTTGGTAAAGCAGCACAGCTTTGTAGAATAGAGGAGAGTCTCATATAAAGTATTGACTTTCTACCAATTCATTTGGGTTCGAAAGTGTCATCATCTTCTAAATATTGTAATAGTTTCAAACTTATCTCGTATTAATAATAATAATAATTAAAAAAAATAATCCGATAAGATAAGAACTGAAAAAGCCCACAGCAAGCACGCATTAGCTGTTCTGTGGCACAATCGCAAATTTCCGAATCCAGCCCGAAGCGCCACGACGCTACTAGAAAATCGCCACGTCATCACATAAAGTAAATATCGGAAATATCTAGAGGAAGGTTATAAATATCTGTCACAGCGAGCAAGTTCCGAAGATTTCTGCATTCGATCAACTTGGAAGCTTAAAGAGCAGCAAAGCGCAAAAAACCAAACCAGCAGAAAAAGGCTGAAATTACAGTCGACCGGAAGGTGAAAACGCTAAAACGCGTCTGAAATTGTCGTATTGATTCGAGAAAGTCGAAGACTGCGGTGCCGATCGGGAAGTTTGTGAGTGGGACTCGTCGATTCGGGAGATTCTGAGGTTGGTTGAGTTTGAGCAGTTTTGATTTCGTTTCCTTTTTGCTTGATGAAATTGTCTGTTTCGTTTCTCGATTAGGGTTTCTAAGCTTAAACTTGTTGGGGAAGGAGGATTTTGTTTCCATTTTTTTTTTTTTTTTTGGGTTGAAATTTGGGGTTTTGAGTAATTGGGTTTTATGAAACTAGGGTTTTTGGATTTGGGATTTTCCTTTTTCCCGGGCTACCACGATTGCGTATCTGTTTTGTAAATTGATGTATTATCGGCGGACTTGGGACCCAAGAAAGCTGAGAGTCCTGGTTCAATTTTGATTTGGTGTATTTGGGTCTTGTCTGTGTATAAAACCCAAAGGTGCTTGTGTGATTGGAACCGCAATTTTCTTTCGAGAATTTCTGTACCCAGTTCTTAATGGAATTTGGTTCGTGTTTACTGCAGATTGTGCATAATAGAGGAGCA
